# Characterization and phylogenetic analysis of the complete chloroplast genome sequence of *Phalaenopsis deliciosa* (Rchb. f. 1854)

**DOI:** 10.1080/23802359.2024.2420842

**Published:** 2024-11-01

**Authors:** Peizhang Chen, Zhongyang Zhang, Xiqiang Song, Zhe Zhang

**Affiliations:** a School of Tropical Agriculture and Forestry, Hainan University, Haikou, China; b Sanya Institute of Breeding and Multiplication, Hainan University, Sanya, China

**Keywords:** *Phalaenopsis*, chloroplast, genome assembly, phylogenetic analysis

## Abstract

*Phalaenopsis deliciosa* (Rchb. f.), an ornamental orchid known for vibrant flowers, has a 148,090 bp chloroplast genome with 36.78% GC content. It includes an 85,241 bp large single-copy (LSC) region, an 11,649 bp small single-copy (SSC) region, and two 13,800 bp inverted repeats (IRs), encoding 122 genes (76 protein-coding, 38 tRNA, and 8 rRNA). This genome data refines the *Phalaenopsis* gene database and supports research on phylogeny and molecular breeding.

## Introduction

**Figure 1.**
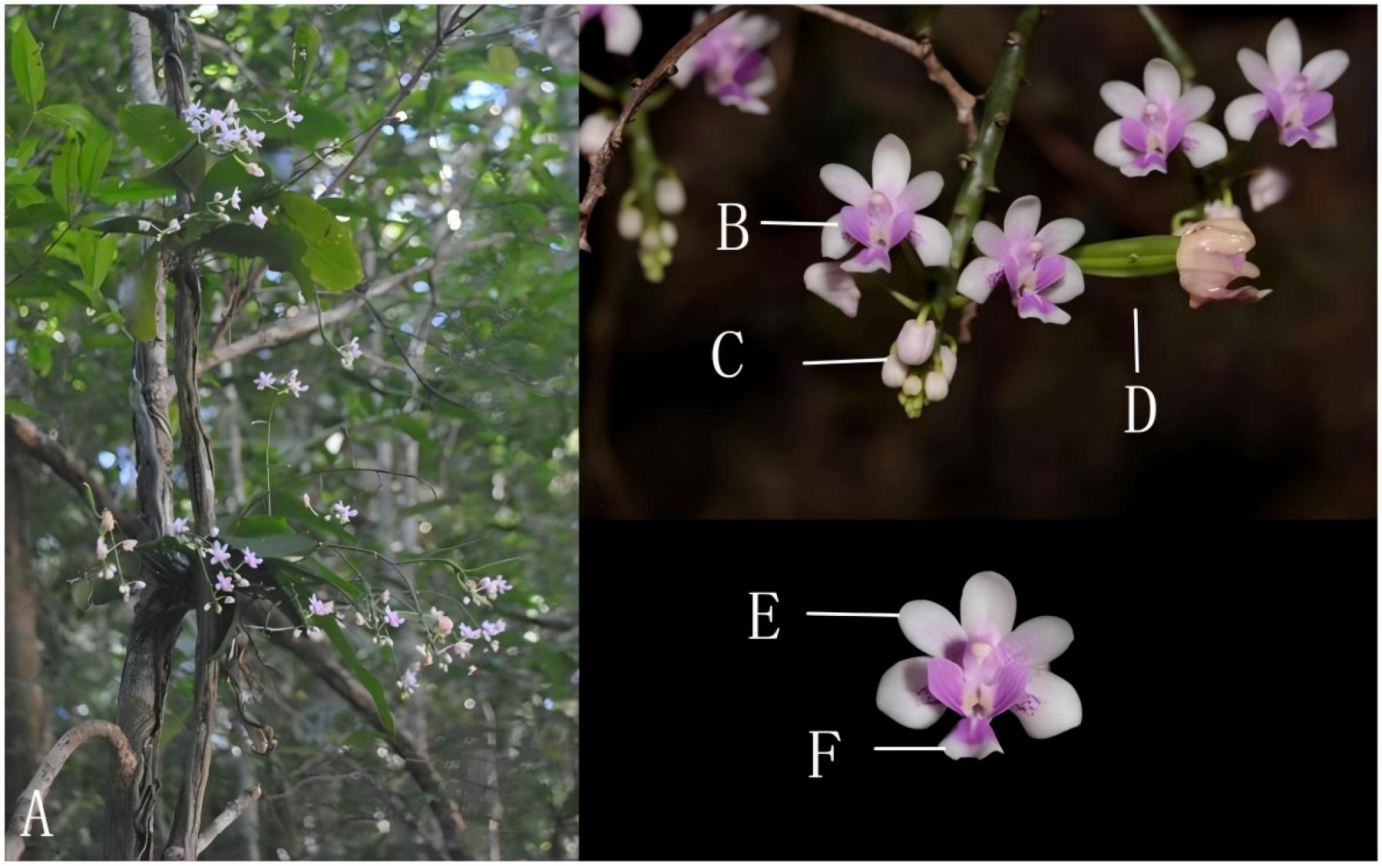
*Phalaenopsis deliciosa*’s plant habitat and flower: (A) plant habitat and entire plant in full bloom; (B) flowers in full bloom; (C) flower bud; (D) fruit; (E) petal; (F) labellum. The characteristic morphological feature is the sepals and petals white with lavender markings, tabellum light purplish red, with a short conical spur and the fruits are capsules. Photographed by Zhongyang Zhang.

*Phalaenopsis* (Vandeae, Orchidaceae) comprises more than 70 species worldwide, distributed in East Asia, South Asia, Southeast Asia, and North Oceania (Chen et al. 2009). Morphological and genetic evidence suggests that *Phalaenopsis* can be divided into four subgenera: *Phalaenopsis*, *Parishianae*, *Hygrouchilus*, and *Ornithochilus* (Li et al. 2016). Among them, the classification of the subgenus *Parishianae* is more complicated, containing four sections: *Parishianae*, *Esmeralda*, *Aphyllae*, and *Deliciosae* (Liu et al. 2020). More sample material is needed to refine the classification of subgenus *Parishianae. Phalaenopsis deliciosa* (Rchb. f. 1854) is distributed in South Asia and Southeast Asia, growing on tree trunks or valley rocks in mountain forests at altitudes of 450–1100 m. *P. deliciosa* was once considered a separate genus, *Kingidium*, but molecular evidence suggested that it should be assigned to the subgenus *Parishianae* of *Phalaenopsis* (Padolina et al. 2005). The habitat and basic characteristics of *P. deliciosa* are shown in Figure 1.

The chloroplast genome is crucial as it plays a key role in photosynthesis, which directly affects plant growth and development. Moreover, chloroplast genomes are often used in phylogenetic studies due to their relatively conserved nature (Jansen et al. 2007). This study aims to sequence and annotate the chloroplast genome of *P. deliciosa*, to explore the differences between its genes and other species within the genus *Phalaenopsis*, and to provide data support for clarifying the phylogenetic relationships under the genus *Phalaenopsis*, as well as the influence of geological history on species differentiation.

## Materials and methods

With the approval of the Hainan Tropical Rainforest National Park Authority, Bawangling Branch Office, the fresh leaves of *P. deliciosa* were collected from Bawangling region of the Hainan Tropical Rainforest National Park, China (19°4′35″N, 109°13′12″E). A voucher specimen was kept at the Teaching Herbarium of the College of Forestry, Hainan University (Zhang Zhe, 107189517@qq.com, number: HUFB2022717). The annotated chloroplast genome was deposited to GenBank under the accession number: OM792977.

Second-generation sequencing technology was utilized for library construction and sequencing of total DNA extracted from plant leaves. The acquired data were subjected to quality filtering to obtain clean data. The total chloroplast genome was extracted from dry leaves using a modified CTAB protocol and sequenced using next-generation sequencing with the Illumina NovaSeq 6000 (San Diego, CA). The genome sequences were screened and assembled with GetOrganelle. The raw data have been submitted to the NCBI Sequence Read Archive (SRA) database under the accession number SRR18136583.

To evaluate the evolutionary relationships, 13 chloroplast genome sequences including four complete chloroplast genomes of Orchidaceae were used. A maximum-likelihood (ML) tree was constructed using MEGA 11 with 1000 bootstrap replicates, applying the Kimura 2-parameter model of nucleotide substitution (Tamura et al. 2021).

To verify the accuracy of the assembly, trimmed raw sequence data were mapped to the assembled chloroplast genome (Supplementary Figure 1). The cis-spliced genes and one trans-spliced gene were verified to be corrected and annotated using CPGview (Supplementary Figure 2).

## Results

**Figure 2.**
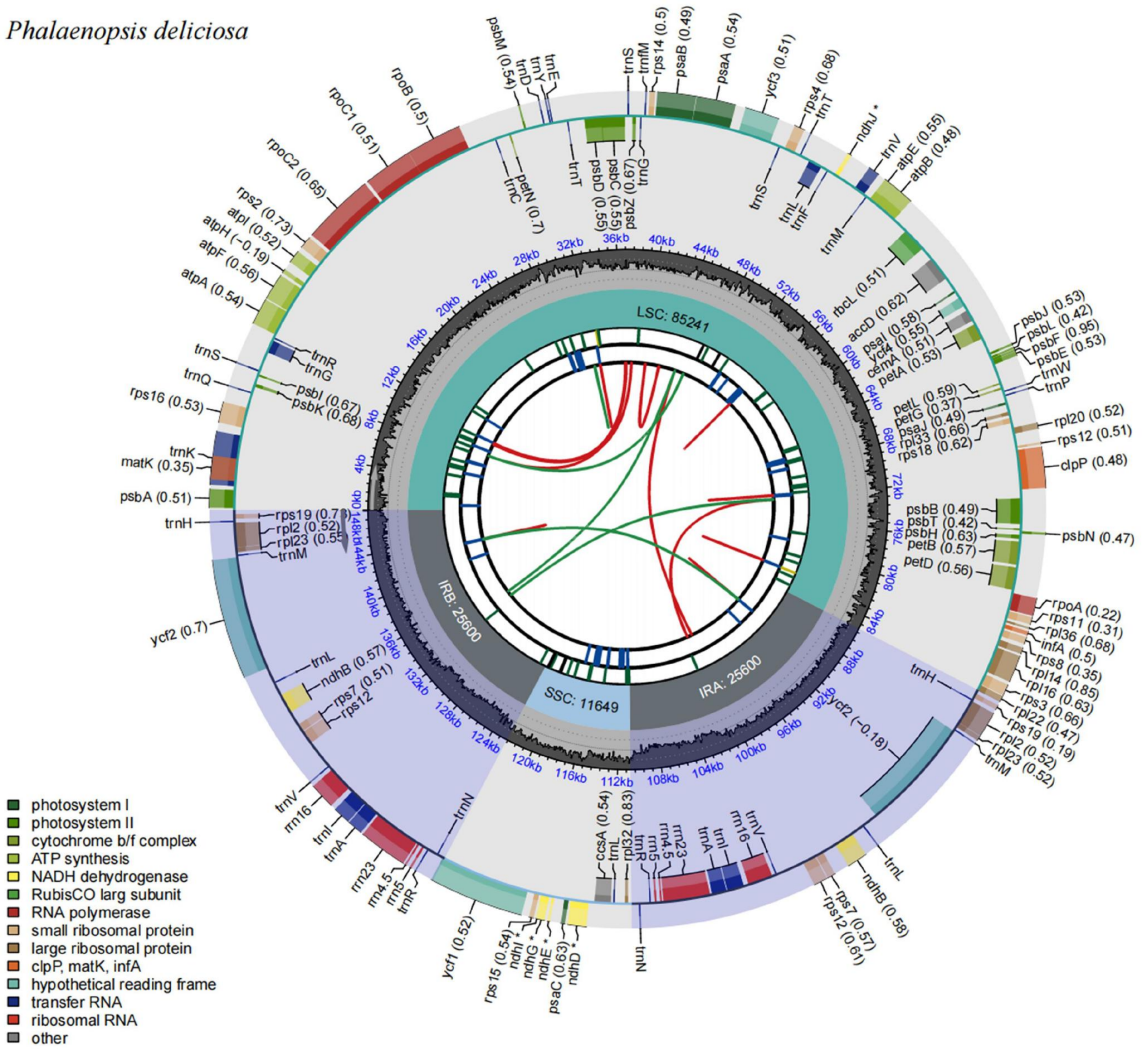
The schematic map presents the overall features of the chloroplast genome. The species name is displayed in the top left corner. By default, the map contains six tracks. From the center outward, the first track displays the dispersed repeats. The dispersed repeats consist of direct (D) and palindromic (P) repeats, depicted with red and green arcs. The second track displays long tandem repeats as short blue bars. The third track displays short tandem repeats or microsatellite sequences as short bars in various colors. The colors, the types of repeats they represent, and their descriptions are as follows: black: c (complex repeat); green: p1 (repeat unit size = 1); yellow: p2 (repeat unit size = 2); purple: p3 (repeat unit size = 3); blue: p4 (repeat unit size = 4); orange: p5 (repeat unit size = 5); red: p6 (repeat unit size = 6). The small single-copy (SSC), inverted repeat (IRa and IRb), and large single-copy (LSC) regions are displayed on the fourth track. The GC content across the genome is plotted on the fifth track. The base frequency at each site along the genome is displayed between the fourth and fifth tracks. The genes are displayed on the sixth track. The optional codon usage bias is indicated in parentheses after the gene name. Genes are color-coded based on their functional classification. The transcription directions for the inner and outer genes are clockwise and counterclockwise, respectively. The functional classification of the genes is displayed in the bottom left corner.

The chloroplast genome of *Phalaenopsis deliciosa* was found to be 148,090 base pairs (bp) in length with an overall GC content of 36.78%. The genome is composed of a large single-copy (LSC) region of 85,241 bp, a small single-copy (SSC) region of 11,649 bp, and two inverted repeat (IR) regions each measuring 13,800 bp (Figure 2). The genome contains a total of 122 genes, including 76 protein-coding genes, 38 transfer RNA (tRNA) genes, and eight ribosomal RNA (rRNA) genes. Ten cis-spliced genes (*rps16*, *atpF*, *rpoC1*, *ycf3*, *clpP*, *petB*, *petD*, *rpl16*, *rpl2*, *ndhB*, and *ycf3*) were verified to be corrected and annotated with multiple sequence alignment (Supplementary Figure 2).

**Figure 3.**
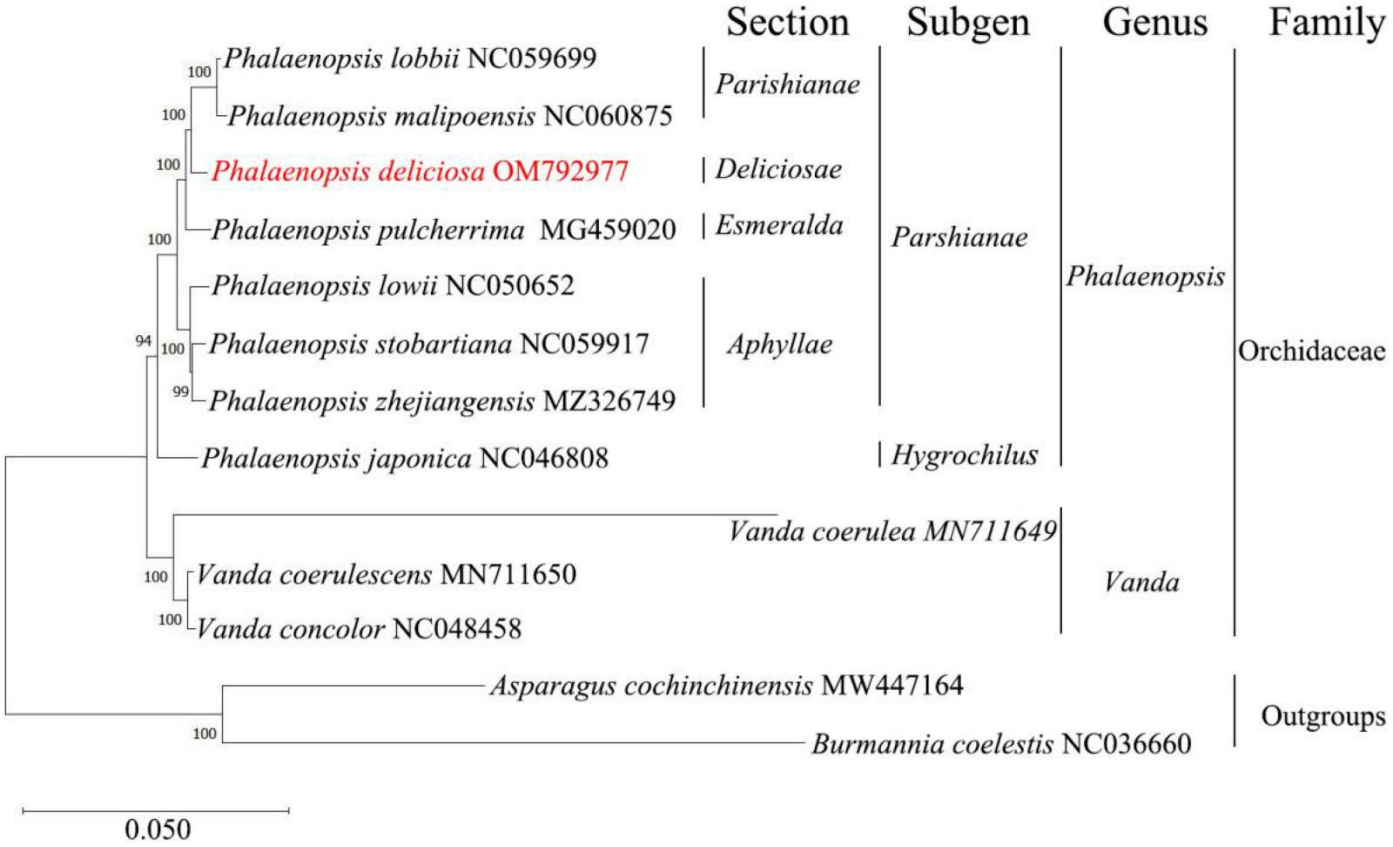
Maximum-likelihood tree based on the chloroplast gene sequences of *P. deliciosa* and 12 other species. The bootstrap values are shown on the nodes, and the species and GenBank accession number are shown at the end of each branch. The 13 chloroplast genome sequences are derived from the following records: *P. lobbii* NC059699 ((H. G. Reichenbach) H. R. Sweet. 1980), *P. malipoensis* NC060875 (Z.J.Liu & S.C.Chen. 2005), *P. deliciosa* OM792977 (Rchb. f. 1854), *P. pulcherrima* MG459020 ((Lindl.) J. J. Sm. 1933), *P. lowii* NC050652 (Rchb.f. 1862), *P. stobartiana* NC059917 (Rchb. f. 1877), *P. zhejiangensis* MZ326749 ((Z.H.Tsi) Schuit. 2012), *P. japonica* NC046808 ((Rchb.f.) Kocyan & Schuit. 2014), *V. coerulea* MN711649 (Griff. ex Lindl. 1847), *V. coerulescens* MN711650 (Griffith. 1851), and *V. concolor* NC048458 (Blume. 1849). *A. cochinchinensis* MW447164 (Lour. 1908) and *B. coelestis* NC036660 (D. Don. 1825) were used as outgroup taxa. Scale bar refers to a phylogenetic distance of 0.050 nucleotide substitutions per site.

The alignment and ML tree construction revealed that the sections *Deliciosae*, *Parishianae*, and *Esmeralda* form a closely related clade, whereas section *Aphyllae* forms a distinct separate branch (Figure 3). This close relationship within the clades was supported by high bootstrap values, demonstrating a robust phylogenetic framework.

## Discussion and conclusions

*P. deliciosa*, as a representative species of the *Deliciosae* section, occupies a critical position within the phylogenetic tree, highlighting its significant evolutionary divergence from other sections. In contrast to the Parishianae section (including species such as *Phalaenopsis lobbii* and *Phalaenopsis malipoensis*), *P. deliciosa* diverged at an earlier point, suggesting that this species experienced unique genetic drift or adaptive evolution, resulting in a distinct evolutionary trajectory.

In the *Phalaenopsis*, the *ndh* genes are predominantly either lost or have become pseudogenes (Lin et al. 2017). However, in *P. deliciosa*, these genes (ndhE, ndhD, ndhG, ndhI, and ndhB) are fully preserved which suggests that *P. deliciosa* has likely been subject to relatively conservative selective pressures during its evolution, maintaining functional dependence on the non-photochemical electron transport pathway.

The distinctiveness of the *Deliciosae* section may be attributed to ecological niche specialization or geographic isolation. Geographic isolation frequently reduces gene flow between populations, ultimately driving speciation (Losos and Glor 1998). Consistent with previous research, the uniqueness of *P. deliciosa* may arise from adaptations to specific habitats or microclimates, such as humid tropical conditions or variations in altitude, which have contributed to its evolutionary differentiation from other species (Chase et al. 2003).

In conclusion, this study has contributed to chloroplast genome resources for the family Orchidaceae and the genus Phalaenopsis. These advancements are expected to facilitate future genetic investigations aimed at the conservation and exploitation of *P. deliciosa*. This will play an important role in the identification of genus *Phalaenopsis* species and population genetic studies.

## Supplementary Material

The cis and trans gene.pdf

Coverage depth.pdf

## Data Availability

The genome sequence data that support the findings of this study are available in GenBank of NCBI (http://www.ncbi.nlm.nih.gov/) under the accession no. OM792977. The associated BioProject, BioSample, and SRA numbers are PRJNA810277, SAMN26237615, and SRR18136583, respectively.
