# Supplementary material for: Characterization and phylogenetic analysis of the complete chloroplast genome sequence of Phalaenopsis deliciosa (Rchb. f. 1854)
Source: Mitochondrial DNA B Resour. 2024 Nov 1;9(11):1483–6. doi: 10.1080/23802359.2024.2420842 (PMC11536658; doi:10.1080/23802359.2024.2420842)
Supplement: The cis and trans gene.pdf [file TMDN_A_2420842_SM2551.pdf]

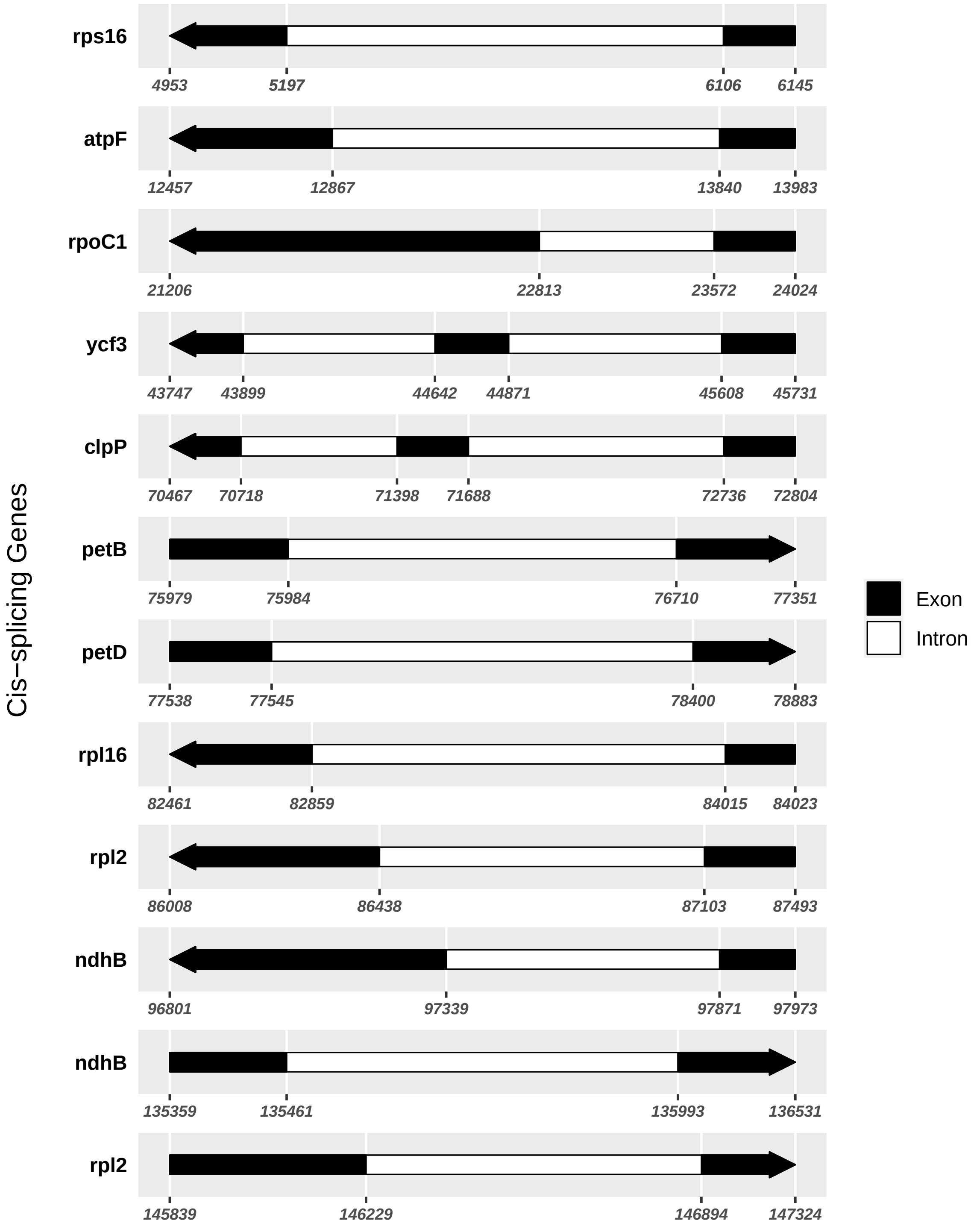

Cis−splicing Genes
rps16
4953
5197
6106
6145
atpF
12457
12867
13840
13983
rpoC1
21206
22813
23572
24024
ycf3
43747
43899
44642
44871
45608
45731
clpP
70467
70718
71398
71688
72736
72804
petB
75979
75984
76710
77351
petD
77538
77545
78400
78883
rpl16
82461
82859
84015
84023
rpl2
86008
86438
87103
87493
ndhB
96801
97339
97871
97973
ndhB
135359
135461
135993
136531
rpl2
145839
146229
146894
147324
Exon
Intron

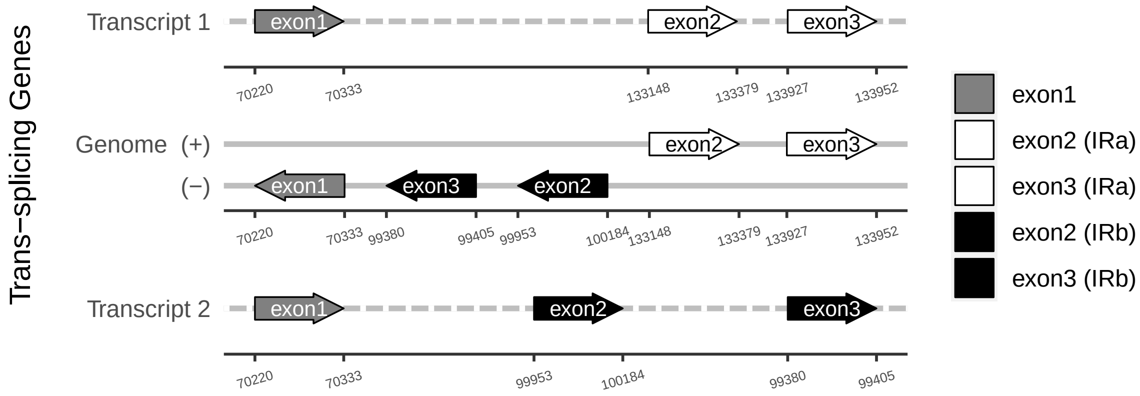

Supplementary Figure 2. Schematic map of the cis-splicing genes (A) and trans -splicing gene (B) in the plastome of *Phalaenopsis deliciosa*. The exons are shown in black; the introns are shown in white for (A). The arrow indicates the sense direction of the gene. The map was generated using CPGview.
