# Supplementary material for: Characterization and phylogenetic analysis of the complete chloroplast genome sequence of Phalaenopsis deliciosa (Rchb. f. 1854)
Source: Mitochondrial DNA B Resour. 2024 Nov 1;9(11):1483–6. doi: 10.1080/23802359.2024.2420842 (PMC11536658; doi:10.1080/23802359.2024.2420842)
Supplement: Coverage depth.pdf [file TMDN_A_2420842_SM2550.pdf]

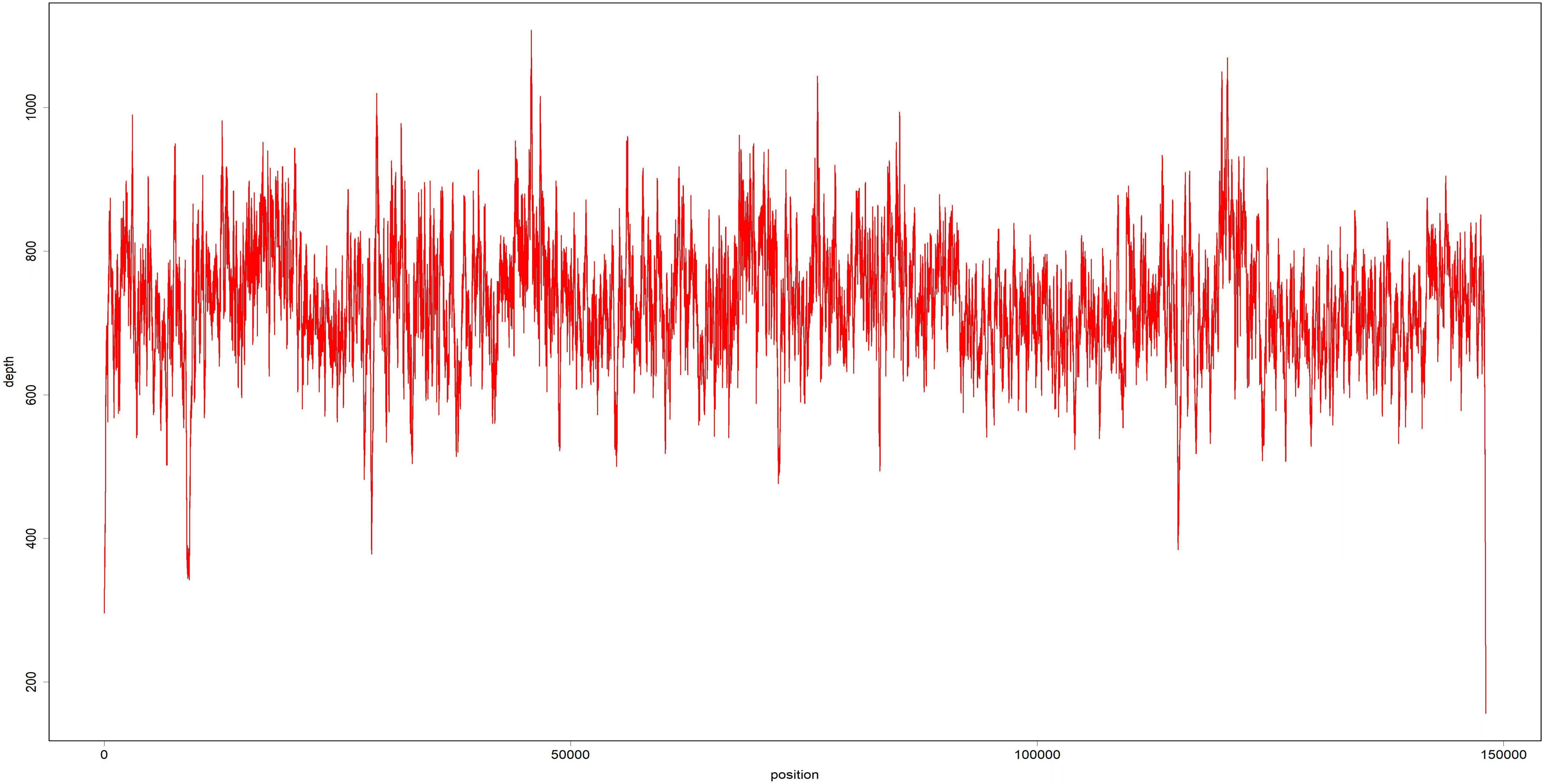

Supplementary Figure 1. Coverage depth figure of the *Phalaenopsis deliciosa* plastome . The horizontal coordinate is the base of the plastome and the vertical coordinate is the depth of sequencing corresponding to that base.
